# Electrochemical Synthesis of Core–Shell-Structured NbC–Fe Composite Powder for Enforcement in Low-Carbon Steel

**DOI:** 10.3390/ma10111257

**Published:** 2017-11-01

**Authors:** Hongmei Li, Qiushi Song, Qian Xu, Ying Chen, Liang Xu, Tiannan Man

**Affiliations:** 1 Liaoning Key Laboratory for Metallurgical Sensor and Technology, School of Metallurgy, Northeastern University, Shenyang 110819, China; hmli2011@126.com (H.L.); chenyinghappyday@163.com (Y.C.); liangxu_neu@163.com (L.X.); 2 State Key Laboratory of Advanced Special Steel, School of Materials Science and Engineering, Shanghai University, Shanghai 200072, China; 3 School of Materials Science and Engineering, Northeastern University, Shenyang 110819, China; manxiaoxiao165@163.com

**Keywords:** NbC–Fe composite, core–shell-structured, electrochemical reduction, molten chloride, cast

## Abstract

An NbC–Fe composite powder was synthesized from an Nb_2_O_5_/Fe/C mixture by electrochemical reduction and subsequent carbonization in molten CaCl_2_–NaCl. The composite has a core–shell structure, in which NbC acts as the cores distributing in the Fe matrix. A strong bonding between NbC and Fe is benefit from the core–shell structure. The sintering and electrochemical reduction processes were investigated to probe the mechanism for the reactions. The results show that NbC particles about several nanometers were embraced by the Fe shell to form a composite about 100 nm in size. This featured structure can feasibly improve the wettability and sinterability of NbC as well as the uniform distribution of the carbide in the cast steel. By adding the composite into steel in the casting process, the grain size of the casted steel was markedly deceased from 1 mm to 500 μm on average, favoring the hardening of the casted steel.

## 1. Introduction

Niobium carbide (NbC) is a non-oxide ceramic material that has been widely used as cutting tools, protective coating for instruments, structural materials, magnetic and electric components because of its excellent chemical and physical properties, such as high melting point (3610 °C), good thermal stability, chemical inertness, low friction coefficient and high hardness (1800 HV) [1,2,3,4]. In particular, NbC powder is used as an additive into other metals or alloys to strengthen the mechanical properties of these materials [4,5,6,7]. The composites consisting of metal and ceramic particle reinforcements play an essential role in the production of cost-competitive and environment-friendly materials [8,9,10]. An addition of ceramic particles can guarantee both hardness and wear resistance of the matrix. Meanwhile, the properties of the matrix, such as ductility and plasticity are inherited [11,12,13]. However, the compatibility is not satisfactory when carbide particles are added into metals because of the low wettability [14]. The products derived usually suffer from the problems of contaminated interfaces and weak bonding between the additives and the matrix [15]. Recently, the introduction of nano-sized carbide particles into a metal matrix to form a core–shell structure has drawn much scientific attention [16,17]. It has been pointed out that the insufficient characteristics of individual carbides can be modified if they are backed by a relatively ductile metal prior to acting as an additive [18], since few challenges exist for the mutual dissolution between metals. 

Conventionally, the metallic matrix-carbide composites are produced via power metallurgy, in which the powder of refractory carbides (such as TiC, WC, and NbC) is adopted as a carbon source, and high purity metals (Ni, Fe, and Cu) as the matrix, experiencing the processes of mixing, ball milling, and vacuum-sintering [19,20,21,22]. However, the severe aggregation of the carbide particles derived from the defects on the surfaces of the products is inevitable. Additionally, energy-intensive issues of high working temperature are involved.

The objective of this study was to prepare a nano-sized NbC–Fe powder in a relatively moderate and convenient way. A novel method was attempted to produce a kind of NbC–Fe composite powder using molten salt electrolysis, in which NbC was selected as a modifier in an Fe matrix to generate a core–shell structure. Nano-sized NbC was synthesized via electrochemical reduction of an Nb_2_O_5_/C/Fe precursor and in-situ carbonization in this precursor. Subsequently, the carbide formed was embraced by Fe previously mixed in the pellet. The phase and microstructure evolution during the reaction was investigated. Finally, the NbC–Fe nano-sized particles were added into a casted steel to test its distribution and wettability.

## 2. Materials and Methods

All starting materials were of analytical grade and purchased from Sinopharm Chemical Reagent Co., Ltd. (Shanghai, China). The powder of Nb_2_O_5_, Fe, and carbon powder was ball-milled with a molar ratio of 2:10:3 (Nb/Fe/C) in ethanol for 1 h. Then, 1.0 g of the mixtures was pressed into a cylindrical pellet (15 mm in diameter and ~1.5 mm in thickness) under a pressure load of 10 MPa, followed by a sintering process in an argon atmosphere at 1000 °C for 3 h to gain enough mechanical strength for further electrolysis. The sintered pellet was attached to a stainless steel wire to serve as the cathode, and a high-density graphite rod (13 mm in diameter, 70 mm in length) was employed as the anode.

A eutectic mixture of CaCl_2_–NaCl (70 wt % CaCl_2_, 30 wt % NaCl) was placed into an alumina crucible and dehydrated for 24 h at 300 °C, which was subsequently heated up to 900 °C. Pre-electrolysis was carried out between two graphite rods at 2.5 V for 0.5 h to remove moisture and other possible impurities [23,24,25]. Then, the assembled sintered pellet was immersed into the melt to replace a graphite rod. The electrochemical experiment was conducted at a constant voltage of 3.0 V with a WYJ 40 A 15 V power supply in the atmosphere of high purity argon. After the termination of electrochemical reduction, the solidified salt remaining in the sample was ultrasonically washed with distilled water and ethanol repeatedly, and dried for characterization. Phase composition of the obtained sample was determined via a D/Max-2500PC X-ray diffractometer (XRD) with Cu Kα radiation, and the morphology and structure were characterized via a JSM-6360L V scanning electron microscope (SEM) equipped with energy-dispersive X-ray (EDS) and a JEM-2010 transmission electron microscope (TEM).

To exemplify the improvement of NbC–Fe in comparison with NbC, both the composite and NbC also obtained via electrolysis in molten salt were added to the low-carbon steel. The NbC–Fe composite or NbC powder was packed with iron foil paper, and put into a MgCl_2_ crucible together with S20C low-carbon steel ingot. The ingots were melted using a vacuum induction melting furnace at 1600 °C for 2 min in an inert argon atmosphere. The liquid in the crucible was stirred mechanically to ensure the uniformity of composition. The samples were then cooled down gradually in the crucible and cut into certain size (20 mm in diameter and 25 mm in height). Hardness of the casted samples was measured using a Vickers hardness tester (HV). The microstructure of the specimens was detected via SEM, and the texture was observed with a metallographic microscope after successive operations of mechanical polishing and corrosion with 4% nitric acid alcohol. 

## 3. Results and Discussion

### 3.1. Analysis of Sintered Pellet

**Figure 1:**
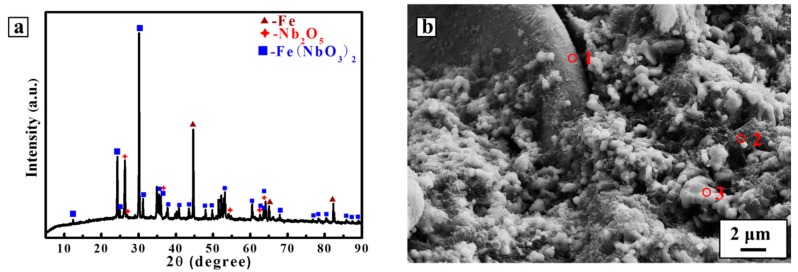
(**a**) X-ray diffractometer (XRD) pattern and (**b**) scanning electron microscope (SEM) image of the Nb_2_O_5_/Fe/C composite pellet sintered at 1000 °C for 3 h.

Figure 1a shows the XRD pattern of the pellet sintered at 1000 °C for 3 h in argon. The peaks of Fe(NbO_3_)_2_ are the most obvious ones; meanwhile, Nb_2_O_5_ and Fe can be detected, whereas the peaks of the carbon additive were not observed because of its amorphous structure. The predominant appearance of Fe(NbO_3_)_2_ implies the existence of Fe_2_O_3_ in the Fe source, and chemical reaction occurred between the Fe species in the sintering process, which subsequently combined Nb_2_O_5_ to generate Fe(NbO_3_)_2_, as indicated in Equations (1) and (2). This phenomenon may not be a significant issue since the oxide phase would be electrochemically decomposed to metallic Fe and Nb during the reduction. Moreover, the nucleation and growth of Fe from Fe(NbO_3_)_2_ during the reduction is available for the formation of nano-sized NbC–Fe particles, since the working temperature for reduction is far lower than the melting point of Fe.

(1) $$Fe_{2}O_{3}+C=2FeO+CO\;\Delta G^{\theta }(1000{}^{\circ}C)=-94.709\;kJ/mol$$

(2) $$FeO+Nb_{2}O_{5}=Fe{\left(NbO_{3}\right)}_{2}$$

Figure 1b shows the SEM image of the sintered pellet with the co-existence of Fe and Fe_2_O_3_ as the source of Fe. Most of the particles aggregate tightly and interconnect with each other after the sintering process. EDS analysis was carried out on various particles, as shown in Table 1. There is a bulk particle much larger than the others in the top left corner of the image, of which the contents of Fe and Nb are about 41% and 3%, respectively. This bulk particle should be Fe with the combination of some niobium oxide during the sintering process. The carbon powder adopted in this study is about 100 nm in size, as indicated by Point 2. Another kind of particle about 1–2 μm in size, as indicated by Point 3, is, according to the EDS analysis in Table 1, mainly composed of Nb_2_O_5_ and Fe(NbO_3_)_2_.

### 3.2. The Reaction Pathway for Electrochemical Reduction and Carbonization

**Figure 2:**
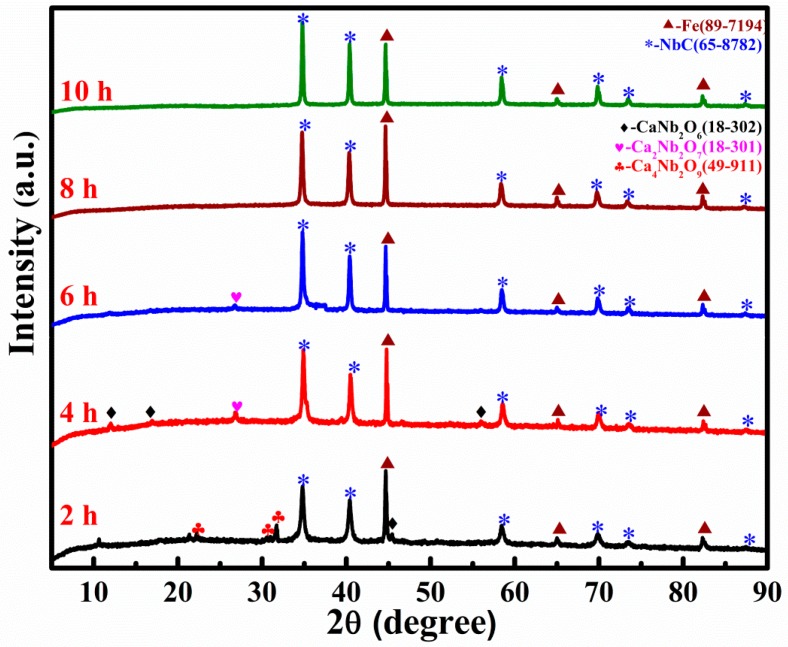
XRD patterns of the samples electrochemically reduced from the Nb_2_O_5_/Fe/carbon composite pellets under 3.0 V for various durations at 900 °C.

In order to investigate the pathway of reduction and subsequent carbonization, the experiment was terminated to obtain completely and incompletely reduced samples. Figure 2 shows the XRD patterns of the samples reduced for various durations. After 2 h of electrolytic reduction, NbC became a predominant phase besides Fe, and a kind of calcium niobate, Ca_4_Nb_2_O_9_, also appeared. This means that Nb-involved compounds such as Nb_2_O_5_ and Ca_4_Nb_2_O_9_ were reduced to Nb, which was subsequently carbonized. At the beginning of the reaction, Ca^2+^ started to penetrate into the pellet and reacted with the unreduced Nb_2_O_5_ to form a more stable phase of calcium niobate. With the prolonging of reaction time, Ca_4_Nb_2_O_9_ was electrochemically reduced to sub-niobate phases of CaNb_2_O_6_ and Ca_2_Nb_2_O_7_, then Ca^2+^ and O^2−^ ions were released into the melt. Meanwhile, NbC–Fe was continuously formed with the expense of Fe and the reduced Nb. Consequently, the diffraction peaks of calcium niobate decreased as the reduction proceeded, and the intensity of the NbC peaks increased in the opposite direction in the XRD patterns. After 10 h of reduction, the diffraction peaks of the calcium niobate completely disappeared. The pellet was only composed of Fe and NbC. Though the phase composition is almost the same for the sample reduced for 8 h, the diffraction peaks for longer electrolysis is sharper and higher, implying a more crystallized structure of the product. Therefore, the reduction was terminated after 10 h to obtain the final product.

**Figure 3:**
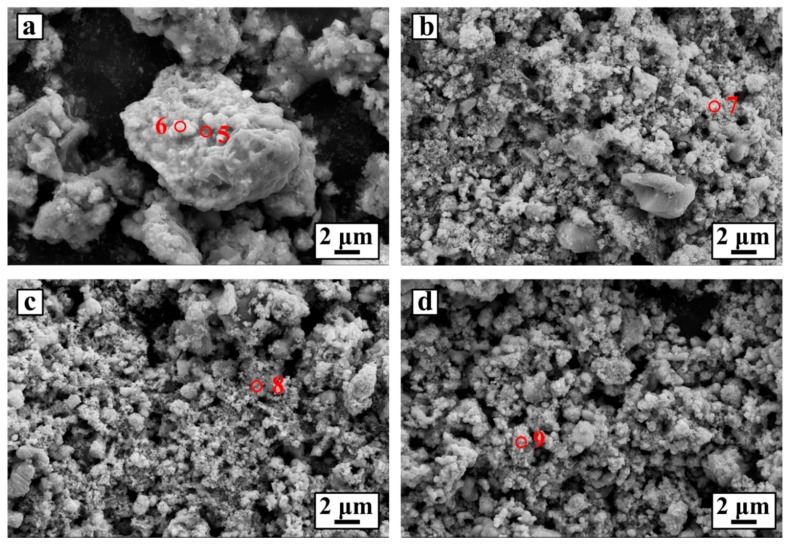
SEM images of the samples electrochemically reduced for (**a**) 2 h; (**b**) 4 h; (**c**) 6 h and (**d**) 8 h under 3.0 V at 900 °C.

Figure 3 shows the SEM images of the samples for various electrolysis durations. The sample that recovered from 2 h of reduction contains particles of about 10 μm, in which Ca_4_Nb_2_O_9_ was gathered with NbC and Fe together. As shown in Figure 3a, there are bright particles and lackluster particles, which can be primarily assigned to Fe particles, and calcium niobate and NbC particles respectively, according to the EDS analysis in Table 2. During the electrochemical reduction, iron combined the niobium-involved compounds to form several secondary particles. This made Fe, niobium compounds and carbon powder aggregate together, which presented an undistinguished morphology of the product in contrast to the morphology of particles in the sintered pellet. When the reaction time reached 4 h, the relatively large particles (in Figure 3a) were decomposed into smaller particles, about less than 4 μm in diameter (in Figure 3b). These particles also contained several co-existing phases of niobate, NbC and Fe based on the EDS and XRD analysis. Furthermore, nano-sized particles that were assigned as NbC also appeared. Figure 3c is the SEM image of the sample after 6 h of reaction. The particles are markedly smaller than those in Figure 3b. This phenomenon shows that calcium niobate was further decomposed, and more niobium carbide was produced. When the reaction time is longer than 6 h, the particles are finer and more homogeneous, since the composition is basically composed of NbC and Fe. Though apparent impurities were not detected after 8 h of reduction, it was found that there was a fair amount of oxygen in the product after 8 h (Table 2), which may be ascribed to the metal (Fe, Nb)–oxygen solid solution as well as the absorbed oxygen on the nano-sized powder during post-treatment. Therefore, the complete reduction was terminated after two extra hours.

**Figure 4:**
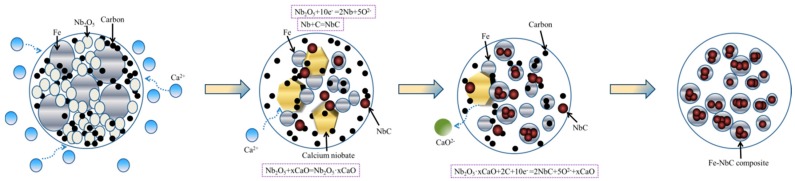
Schematic representation of electrochemical synthesis of the NbC–Fe composite in molten CaCl_2_–NaCl.

Based on the results presented above, the process for electrochemical synthesis of the NbC–Fe composite is summarized and shown schematically in Figure 4. Calcium niobate was generated through the participation of CaO in the melt once the pellet was immersed into the molten salt, as depicted by Equation (3). These reactions may be favorable in thermodynamics, as expressed by Equation (4). Meanwhile, the pentavalent niobium compounds involving Nb_2_O_5_ and Ca_4_Nb_2_O_9_ were electrochemically reduced to niobium, and niobium carbide was obtained via carbonization (Equation (5)). Though the niobium suboxides were not indexed in the XRD patterns, their existence as intermediates in the cathodic pellet can be predicted, as this has been proven by results obtained during direct electro-deoxidation of Nb_2_O_5_ [26].

(3) $$Nb_{2}O_{5}+xCaO=Nb_{2}O_{5}\;•\;xCaO\left(x=1,\;2,\;4\right)$$

(4) $$CaO+Nb_{2}O_{5}=CaNb_{2}O_{6}\;\Delta G^{\theta }\;(900{}^{\circ}C)=-148.283\;kJ/mol$$

(5) $$Nb_{2}O_{5}\;•\;xCaO+2C+10e^{-}=2NbC+5O^{2-}+xCaO\;\left(x=1,\;2,\;4\right)$$

**Figure 5:**
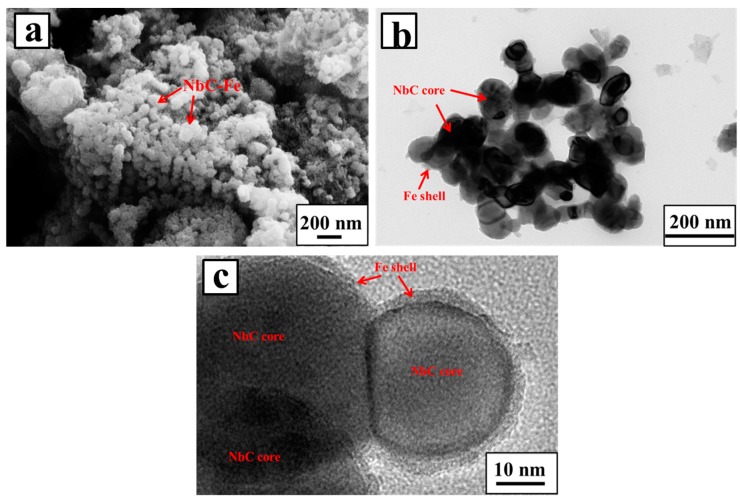
(**a**) SEM image; (**b**) transmission electron microscope (TEM) image; and (**c**) HRTEM image of the samples electrochemically reduced from the Nb_2_O_5_/Fe/carbon composite pellets under 3.0 V for 10 h at 900 °C.

Figure 5a shows the SEM image of the sample reduced for 10 h. NbC–Fe particles with an integrated sphere structure and a homogeneous particle size around 100 nm were successfully obtained. Figure 5b,c present TEM and high-resolution TEM images of the NbC–Fe particles, respectively. The NbC particles are uniformly wrapped in a ductile shell of Fe. The obtained core–shell NbC–Fe structure is helpful for the wettability and sinterability of NbC. Meanwhile, the stability and mechanical properties of the Fe matrix were also improved by the carbide cores.

### 3.3. The Behavior of NbC–Fe in Casted Low-Carbon Steel

**Figure 6:**
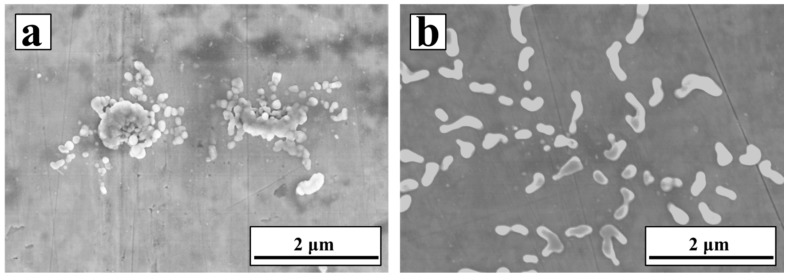
SEM images of the casted steels with different particles (**a**) 0.15 wt % NbC and (**b**) 0.15 wt % NbC in the form of NbC–Fe.

Figure 6a,b shows the SEM images of the low-carbon steels casted with the respective addition of NbC and NbC–Fe having the same amount of NbC (0.15 wt %), in which the NbC powders were electrochemically produced from Nb_2_O_5_ at 900 °C with a constant voltage of 3.0 V for 10 h. It was found that the NbC particles could not disperse uniformly in the casted sample, because of its unsatisfactory wettability with respect to the steel. NbC particles aggregated together seriously in the casted sample. Reversely, the Fe shell outside the carbide effectively improved this deficiency. As shown in Figure 6b, the distribution of the carbide particles is clearly uniform in spite of the fact that they are mixed into the steels under the same conditions. 

**Figure 7:**
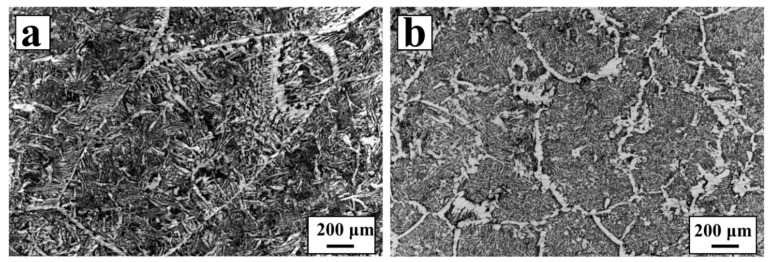

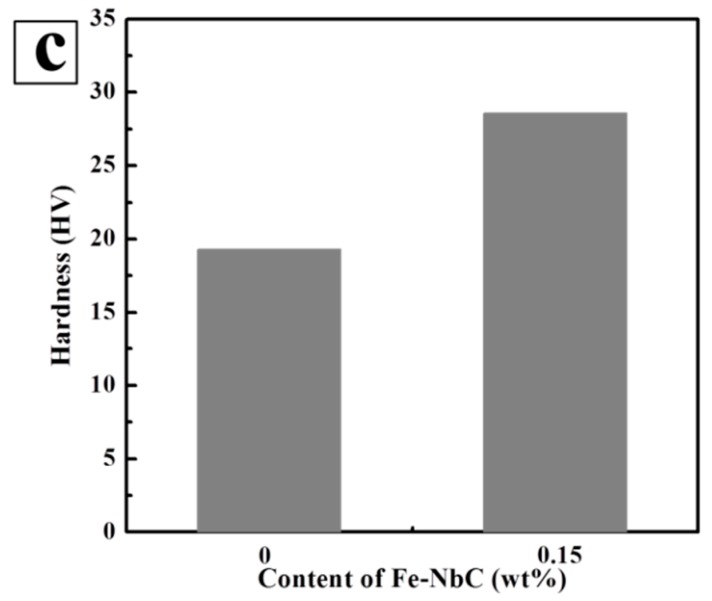
Representative optical micrographs of the cast steels with (**a**) 0 wt % NbC; (**b**) 0.15 wt % NbC in the form of NbC–Fe; and (**c**) hardness of the casted steels.

In order to investigate the effect of the NbC–Fe composite on the metallographic structure of the steel matrix, the specimens were prepared from the casted steel without and with 0.15 wt % NbC in the form of NbC–Fe, which were both mechanically polished and corroded by 4% nitric acid alcohol to observe the texture, as presented in Figure 7a,b. It can be seen that the microstructure of casted steel without NbC is coarse and the grain are more than 1 mm, whereas many extra grain boundaries appears on the same scale, once the NbC–Fe nano-particles were added to the casted steel. The average grain size is less than 500 μm, which is apparently smaller than that in Figure 7a. Therefore, it can be concluded that the addition of the NbC–Fe particles to the casted steel is available for the refinement of the grains. Additionally, the NbC particles reside on the grain boundaries, which is favorable for enforcement of the hardness of the steel. Figure 7c demonstrates the micro-hardness of the samples without and with the mixing of the NbC–Fe composite. The hardness value of the sample with the addition of the NbC–Fe composite is higher than the casted steel without any additives. Though the values of hardness are probably lower than those of the low-carbon steel reported previously, due to the absence of any rolling or heat treatment, the enhancement in hardness also shows the positive effect that the NbC–Fe composite has on the casted steel. The results above show that the addition of NbC–Fe improves its wettability to low-carbon steel, and the enforcement of low-carbon steel has been achieved by modifying its structure.

## 4. Conclusions

Nano-sized NbC–Fe composite particles have been successfully prepared from an Nb_2_O_5_/Fe/C mixture via electrochemical reduction in molten CaCl_2_–NaCl at 900 °C.

Nb_2_O_5_ combined with CaO in the molten salt to generate calcium niobate once the precursor was dipped into the salt. Calcium niobate and niobium oxide were gradually electro-reduced to Nb and finally NbC. The core–shell-structured NbC–Fe composite has the structure of NbC inside as the cores and Fe outside acting as the metallic shell. 

The core–shell structure of NbC–Fe composite can improve the wettability of NbC with respect to low-carbon steel. The effects of the additions of NbC and NbC–Fe nano-particles in the cast steels were investigated. The NbC–Fe composite distributes more uniformly than pure NbC in the casted steels by taking advantage of the Fe shell. The addition of the composite also achieves the decrease of the grain size in the casted steel. The refinement of grains and the aggregation of NbC on the grain boundaries is favorable for the strengthening of the casted steel. 

## Figures and Tables

**Table 1:** Energy-dispersive X-ray (EDS) analyses of Point 1, Point 2, and Point 3 marked in Figure 1b.

Point	Elementary Distribution (at %)			
	C	O	Fe	Nb
1	33.02	22.13	41.67	3.18
2	84.31	10.10	0.69	4.90
3	0	71.48	4.98	23.53

**Table 2:** EDS analyses of Point 5 and Point 6 marked in Figure 3a; Point 7 marked in Figure 3b; Point 8 marked in Figure 3c and Point 9 marked in Figure 3d.

Point	Elementary Distribution (at %)				
	Ca	Nb	C	O	Fe
5	4.17	11.58	35.92	40.02	4.80
6	1.26	8.85	37.18	24.97	26.87
7	0.21	10.70	55.92	12.21	20.43
8	0.14	12.84	42.56	36.28	7.65
9	0.20	18.72	44.17	28.15	8.76
